# Multisystem Disease With Suspected Malignancy: Clinical Management Following Diagnostic Refusal

**DOI:** 10.7759/cureus.103148

**Published:** 2026-02-07

**Authors:** Márcia Moreira Costa, Anabela Andrade, Ana Raquel Dias, Marta Henriques Baptista, Afonso Carvalhal

**Affiliations:** 1 Family Medicine, Unidade Local de Saúde da Região de Aveiro, USF Senhora de Vagos, Vagos, PRT

**Keywords:** continuity of patient care, diagnostic uncertainty, palliative care, patient autonomy, primary health care, shared decision-making

## Abstract

Follow‑up of older adults in primary care often entails clinical challenges that go beyond diagnosis. Comorbidities, functional limitations, and social vulnerabilities require a holistic, person‑centred approach grounded in a continuous and trusting doctor-patient relationship. We present a case of an 88-year-old man followed in Family Medicine consultations, who presented with cervical and abdominal skin lesions evolving over three months. Imaging findings were highly suggestive of disseminated oncologic disease. Two biopsies were performed, but no definitive diagnosis was reached. After a clear clinical explanation regarding diagnostic hypotheses and likely disease progression, the patient refused further aetiological investigations. Respecting the principle of autonomy, the clinical management strategy shifted towards continuous follow‑up, adaptation of the therapeutic plan, and a referral to palliative care was proposed. This case underlines the importance of individualised care and adherence to ethical principles, particularly patient autonomy, when diagnostic investigation is declined. It illustrates how primary care physicians can maintain clinical oversight and deliver quality care when faced with diagnostic uncertainty.

## Introduction

Clinical practice in Family Medicine is grounded in a biopsychosocial approach, where the therapeutic relationship, contextual understanding, and continuity of care are central pillars [1]. When confronted with signs suggestive of serious illness, particularly oncological disease, an aetiological investigation is typically conducted. This usually involves a structured diagnostic work-up, aiming to identify the underlying cause and initiate appropriate treatment. However, when patients decline further investigation, clinical priorities shift toward symptom-oriented management, structured follow-up, shared decision-making, and advance care planning.

Any diagnostic approach must respect the patient’s autonomy and sociocultural context [2]. A refusal to proceed with further diagnostic procedures should not be seen as the end of clinical care, but rather as a prompt to adapt the management plan in order to align with the patient’s values and preferences, within an ethical framework [2-4]. This case illustrates how primary care physicians can ensure meaningful and ongoing follow-up, even in the absence of a definitive diagnosis.

## Case presentation

The patient was an 88-year-old retired man, formerly an electrician, living independently and with no apparent signs of cognitive impairment. Cognitive screening using the Mini-Mental State Examination (MMSE) scored 28/30, and the patient was oriented to person, place, and time. He lived with his wife, who was also functionally independent and had a diagnosis of myelodysplastic syndrome. Their social support network was limited: they had no nearby relatives, and the patient maintained regular phone contact only with one of his two daughters, both of whom lived abroad. He reported managing daily activities autonomously, with no external caregiving support at the time of consultation.

The patient reported no history of smoking or alcohol consumption. His medical history included hypertension, dyslipidaemia, and benign prostatic hyperplasia. His medications included candesartan 16 mg plus hydrochlorothiazide 12.5 mg once daily, atorvastatin 20 mg plus ezetimibe 10 mg once daily, acetylsalicylic acid 100 mg daily, and Serenoa repens.

**Figure 1:**
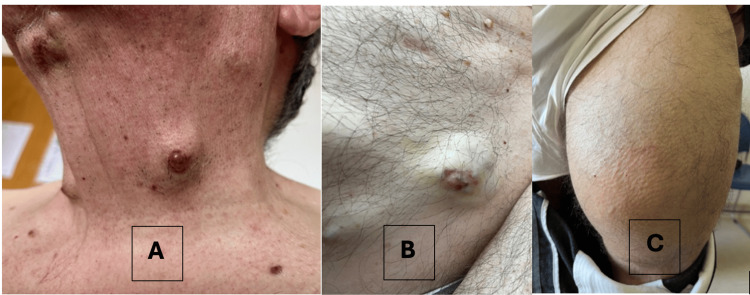
Clinical presentation of cutaneous and subcutaneous lesions. A) Firm, violaceous nodules on the cervical region; B) Subcutaneous lesion with superficial ulceration on the abdominal wall; C) Large, longstanding indurated mass on the anterior aspect of the left thigh.

During a routine Family Medicine consultation, the patient reported the onset of multiple rapidly growing subcutaneous lesions located on the scalp, neck, chest, and abdomen. He mentioned only mild, non-significant weight loss (two kilograms over nine months) and denied fever, anorexia, along with respiratory, gastrointestinal, or genitourinary symptoms. On physical examination, approximately 10 cutaneous nodules were noted on the scalp, neck, chest, and abdominal wall, measuring between 1 and 5 cm in diameter (Figure 1). These were firm, non-tender, violaceous, and in some cases fixed to underlying tissues. One lesion on the abdominal wall presented with superficial ulceration (Figure 1B). A large, longstanding indurated mass measuring approximately 18 × 13 cm was also identified on the anterior aspect of the left thigh (Figure 1C), reportedly unchanged for over two decades. No axillary or cervical lymphadenopathy, hepatosplenomegaly, or peripheral oedema were detected. Vital signs were: BP 112/58 mmHg, BMI 27.3 kg/m², afebrile. Cardiopulmonary and abdominal examinations were unremarkable.

A thoracoabdominopelvic computed tomography (CT) scan and blood tests were requested. Blood tests were performed and showed mild normocytic anaemia with increased red cell distribution width (RDW), along with slightly elevated inflammatory markers, including erythrocyte sedimentation rate and C‑reactive protein. Renal function was preserved, and liver enzymes were within normal limits, except for a mild elevation in lactate dehydrogenase (LDH). Serum protein electrophoresis revealed a diffuse increase in the gamma-globulin fraction without a monoclonal peak, consistent with a possible polyclonal hypergammaglobulinaemia. Tumour marker analysis showed a mildly elevated prostate-specific antigen (PSA) level (total PSA 4.90 ng/mL, free/total PSA ratio 46.9%), while other tumour markers (carcinoembryonic antigen (CEA), CA 19‑9, CA 15‑3, CA 72‑4, alpha-fetoprotein (AFP), and Cyfra 21‑1) were within the reference range (Table 1).

**Table 1:** Summary of selected laboratory results at initial evaluation. Values are presented with corresponding reference ranges. PSA: prostate-specific antigen; RDW: red cell distribution width; ESR: erythrocyte sedimentation rate; CRP: C-reactive protein; LDH: lactate dehydrogenase; CEA: carcinoembryonic antigen; AFP: alpha-fetoprotein.

Test	Result	Reference Range
Hemoglobin (Hb)	12.7 g/dL	13.0–17.0 g/dL
Mean Corpuscular Volume (MCV)	88.9 fL	80–100 fL
Red Cell Distribution Width (RDW)	15.6%	11.5–14.5%
Erythrocyte Sedimentation Rate (ESR)	43 mm/h	<20 mm/h
C-Reactive Protein (CRP)	12.2 mg/L	<5 mg/L
Lactate Dehydrogenase (LDH)	271 U/L	135–225 U/L
Creatinine	0.91 mg/dL	0.70–1.20 mg/dL
Alanine Transaminase (ALT)	29 U/L	<41 U/L
Aspartate Transaminase (AST)	25 U/L	<40 U/L
Total PSA	4.90 ng/mL	<4.00 ng/mL
Free/Total PSA Ratio	46.9%	>25%
CEA	1.3 ng/mL	<5.0 ng/mL
CA 19-9	6.4 U/mL	<37 U/mL
CA 15-3	8.8 U/mL	<25 U/mL
CA 72-4	3.5 U/mL	<6.9 U/mL
Alpha-fetoprotein (AFP)	1.8 ng/mL	<10 ng/mL
Cyfra 21-1	1.0 ng/mL	<3.3 ng/mL
Serum Protein Electrophoresis	Polyclonal increase in gamma globulins	No monoclonal peak

**Figure 2:**
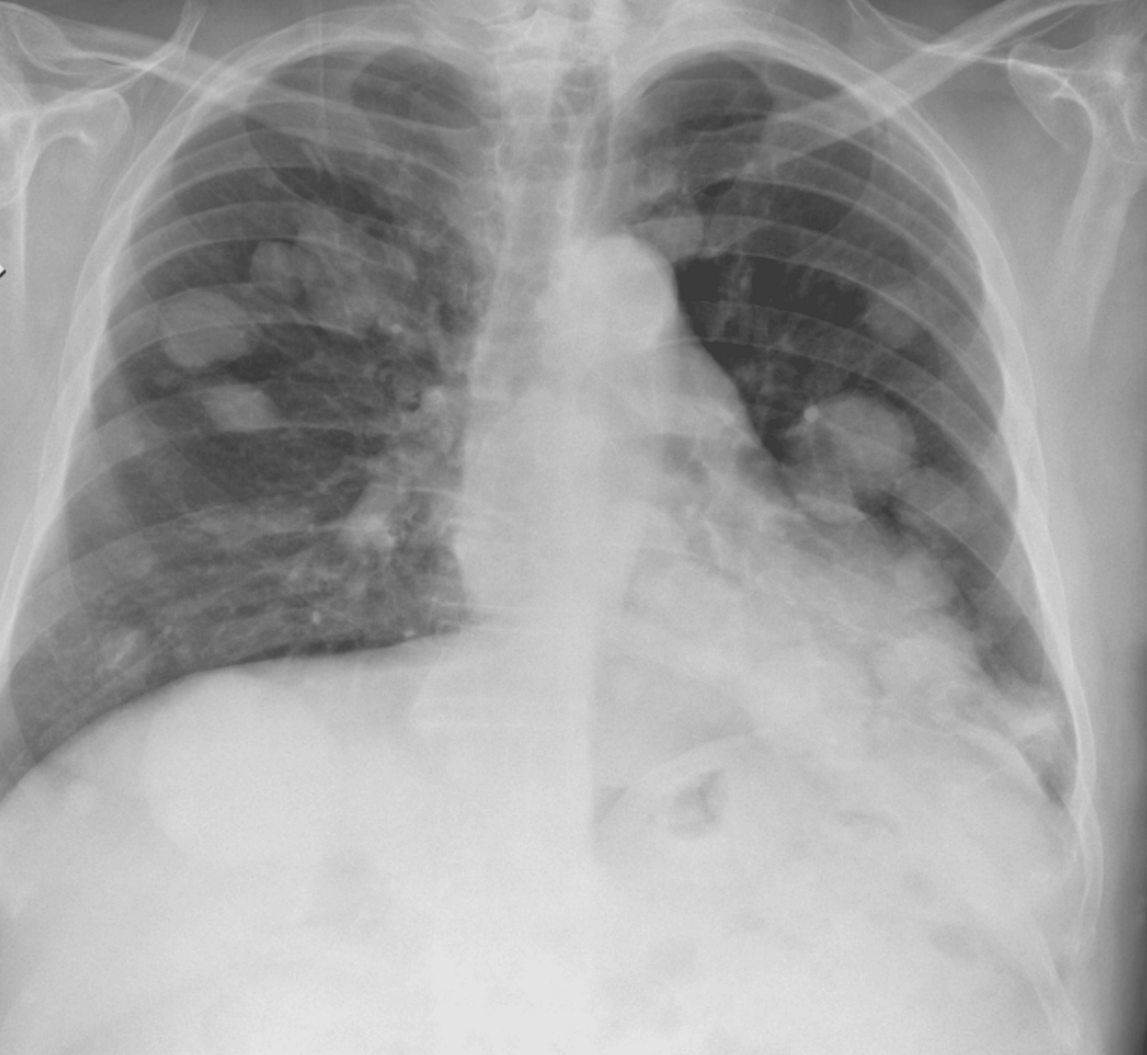
Chest radiograph showing multiple bilateral pulmonary nodules. The radiographic pattern suggests hematogenous spread, raising suspicion for disseminated metastatic disease.

The CT revealed multiple subcutaneous and intramuscular lesions, nodular pulmonary lesions, mediastinal lymphadenopathy, and lytic bone lesions. The thigh mass involved diffuse infiltration of the left quadriceps. These findings were strongly suggestive of disseminated oncologic disease. Additionally, a chest radiograph (Figure 2) showed multiple bilateral nodular opacities with a random distribution pattern. While some nodules displayed well-defined borders, others had ill-defined margins, raising suspicion for hematogenous dissemination. These radiologic findings further supported the hypothesis of widespread metastatic disease. However, the patient was unable to retrieve the CT images from the oncology centre due to logistical limitations; thus, representative CT images could not be included in this report.

The patient was referred to the national oncology centre, where he underwent a multidisciplinary evaluation by the Internal Medicine, Dermatology, and Pulmonology teams. An excisional biopsy of a cervical nodule (Figure 1A) revealed histological features consistent with chondroid syringoma, without evidence of malignancy. Due to the discordance between histological findings and the clinical/imaging context, a second incisional biopsy was performed on an abdominal lesion (Figure 1B), which revealed mucinous adenocarcinoma with extensive necrosis. Immunohistochemical analysis was non-contributory due to lack of viable tissue, and repeat sampling was recommended.

After explaining the likely diagnosis and need for further investigation (including a third biopsy), the patient declined. At a follow-up visit, he confirmed his decision, citing the absence of symptoms and his advanced age. His ability to understand, deliberate, and communicate healthcare decisions was reaffirmed during the consultation, supporting the ethical basis for informed refusal. Respecting his autonomy, a referral for hospital-based palliative care was proposed which he accepted but preferred to postpone scheduling. The clinical course and key milestones are summarized in Table 2.

**Table 2:** Timeline of the Patient’s Clinical Course

Date	Clinical events
April 2025	Onset of multiple cutaneous lesions
July 2025	Primary care follow‑up consultation: mass and cutaneous lesions identified. Laboratory workup and thoraco‑abdominopelvic CT scan requested
August 2025	CT scan revealed multiple cutaneous, muscular, pulmonary, and osseous lesions. Referral to the Oncology Institute.
September 2025	Incisional biopsy of an anterior cervical lesion (05/09/2025): diagnosis of benign chondroid syringoma. Biopsy of an abdominal lesion (26/09/2025): findings suggestive of mucinous adenocarcinoma, without immunohistochemistry
October 2025	Patient declined further investigation; hospital teams proposed palliative care referral
November 2025	Continued follow‑up in primary care

In the primary care setting, a management plan was developed including regular clinical monitoring, functional assessment, and medication review. Advance care planning was initiated, addressing the patient's values, preferences, and potential future treatment limitations. Given the fragile support network and his wife’s health condition, psychosocial support was offered, and home care services and community resources were considered depending on future clinical needs.

## Discussion

This case exemplifies the complexity frequently encountered in primary care, particularly when there is strong clinical suspicion of serious disease but further investigation is limited by patient choice. In such scenarios, a person-centred approach becomes essential, emphasising attentive listening, transparent communication, respect for autonomy, and adaptable care planning [1-3].

The presence of multiple cutaneous and visceral lesions, along with imaging findings highly suggestive of disseminated occult malignancy, warranted further diagnostic investigation. However, the absence of definitive histological confirmation and the patient’s refusal to pursue further testing required a readjustment of the clinical strategy without compromising continuity of care.

The physician’s longstanding relationship with the patient, coupled with an understanding of his personal history, family dynamics, and core values, enabled close and individualised follow-up. Active surveillance, clear and open communication, and the non-coercive proposal of palliative care reflect key principles of Family Medicine as outlined by the World Organization of National Colleges, Academies, and Academic Associations of General Practitioners/Family Physicians (WONCA), including person-centredness, care continuity, and complexity management [1].

While chondroid syringoma is a rare benign cutaneous neoplasm, its isolated identification in the context of widespread disease demands caution in interpretation, particularly when it contradicts clinical and imaging findings. Although malignant variants of chondroid syringoma with infiltrative or metastatic potential have been described, their rarity calls for a broader differential diagnosis, including primary malignancies of non-cutaneous origin, which appears more likely in this case [5-7].

The finding of extensive necrosis and morphological features suggestive of mucinous adenocarcinoma in the second biopsy raised the possibility of an undetected primary malignancy. Although the primary tumour remained unidentified, a gastrointestinal or pancreatic origin could not be excluded. In the context of strong clinical and radiological suspicion of malignancy, the absence of histological confirmation highlights the diagnostic limitations posed by suboptimal sampling. This case therefore underscores the importance of integrating clinical, imaging, and pathological data to support informed, values-based decision-making [2-4,8].

## Conclusions

The absence of a definitive diagnosis in this case may reflect clinical complexity and shared decision-making, rather than a failure of the diagnostic process. Upholding ethical and professional principles, particularly respect for patient autonomy, is essential to building a strong doctor-patient relationship and ensuring appropriate care. Managing clinical uncertainty requires not only technical competence but also a solid ethical foundation and relational skills that support continuity and progressive adaptation of care plans. This case exemplifies how primary care can accommodate diagnostic uncertainty through ethical, person-centred care that preserves patient dignity and supports ongoing reassessment.
